# Therapeutic Strategies for Duchenne Muscular Dystrophy: An Update

**DOI:** 10.3390/genes11080837

**Published:** 2020-07-23

**Authors:** Chengmei Sun, Luoan Shen, Zheng Zhang, Xin Xie

**Affiliations:** 1 Zhejiang University-University of Edinburgh Institute, School of Medicine, Zhejiang University, Haining 314400, China; chengmeisun@intl.zju.edu.cn (C.S.); luoan.18@intl.zju.edu.cn (L.S.); Zheng.18@intl.zju.edu.cn (Z.Z.); 2 Department of Medical Oncology, the Second Affiliated Hospital of Zhejiang University School of Medicine, Zhejiang University, Hangzhou 310009, China

**Keywords:** Duchenne muscular dystrophy, pathogenesis, dystrophin restoration, gene therapy, cell transplantation

## Abstract

Neuromuscular disorders encompass a heterogeneous group of conditions that impair the function of muscles, motor neurons, peripheral nerves, and neuromuscular junctions. Being the most common and most severe type of muscular dystrophy, Duchenne muscular dystrophy (DMD), is caused by mutations in the X-linked *dystrophin* gene. Loss of dystrophin protein leads to recurrent myofiber damage, chronic inflammation, progressive fibrosis, and dysfunction of muscle stem cells. Over the last few years, there has been considerable development of diagnosis and therapeutics for DMD, but current treatments do not cure the disease. Here, we review the current status of DMD pathogenesis and therapy, focusing on mutational spectrum, diagnosis tools, clinical trials, and therapeutic approaches including dystrophin restoration, gene therapy, and myogenic cell transplantation. Furthermore, we present the clinical potential of advanced strategies combining gene editing, cell-based therapy with tissue engineering for the treatment of muscular dystrophy.

## 1. Introduction

Duchenne muscular dystrophy (DMD) is a lethal pediatric muscle disorder, affecting 1 out of 5000 males born worldwide [1]. DMD leads to progressive muscle weakness and wasting, and most patents die by the age of 30 due to cardiorespiratory failure [1]. For decades, scientists have been trying to find effective treatments for this tragic disease. Although there is no absolute cure for DMD, therapies that can delay the onset or slow down the progression of disease have been developed over the past few decades [2].

DMD is a genetically inherited single-gene disorder caused by mutations in the *DMD* gene [2]. Located on the X chromosome, the *DMD* gene is the largest known human gene, consisting of 79 exons which encodes a 3685 amino acids protein called dystrophin [3]. Dystrophin is a cytoskeletal protein that connects the dystrophin-associated protein complex (DAPC) and the intracellular cytoskeleton c-actin [4]. It contains four functional domains: actin-binding amino-terminal domain (ABD), a central rod domain, a cysteine-rich domain, and a carboxy-terminal domain [5]. Given that DMD mutations lead to dystrophin deficiency, therapies that restore dystrophin expression have been developed to meet clinical needs. 

Previous studies of DMD pathogenesis mainly focused on muscle wasting caused by dystrophin deficiency in myofibers, whereas recent findings suggest that DMD may also be a stem cell disease [6]. In DMD patients, muscle stem cells (also called satellite cells, SC) harbor the same mutations as those in muscle fibers. Loss of dystrophin in SCs leads to abnormalities in SC polarity, asymmetric division, and epigenetic regulation, thus contributing to the DMD pathophysiology [6]. Here, we summarize new evidence of DMD pathogenesis and highlight therapies targeting the *DMD* gene defects in both muscle fibers and myogenic stem cells.

## 2. Genetic Pathogenesis of DMD

### 2.1. An Overview of Dystrophin Gene Mutations—Types and Sites

Since the discovery of the *DMD* gene in 1987, many different types of mutations have been identified [7]. In a recent analysis including 7149 DMD patients [8], large mutations (more than one exon affected) were identified in approximately 79% of the patients, in which large deletions account for 68% and large duplications are responsible for the rest 11%. The remaining 21% patients carry small mutations, out of which, half are nonsense mutations. Small deletions, small insertions, and splice sites mutations represent 5%, 2%, 3% of the total patient population, respectively. 

Interestingly, the deleted exons tend to be clustered in the region from exon 43 to 55. Mutations within these hot spot sites occur in roughly 60–65% of the patients. Exon 51 (mutation rate 14%), exon 53 (10.1%), exon 45 (9%), exon 43 (7.5%), and exon 44 (7.1%) are the top five affected exons according to this large-scale study [8]. 

### 2.2. Correlation between Mutations and Disease Severity 

The effects of *DMD* gene mutations are primarily determined by whether the variants disrupt the reading frame downstream of those mutations. In case of out-of-frame mutations, the open reading frame of the *DMD* gene is disrupted, leading to dystrophin deficiency and a severe DMD phenotype. Meanwhile, in-frame mutations preserve the reading frame, generating partially functional dystrophin protein and leading to less severe Becker muscular dystrophy (BMD) disease [9,10,11]. It is worth noticing that BMD phenotypes can be highly variable due to the types of mutations, ranging from asymptomatic to borderline DMD [11]. 

This open reading frame rule, so-called the Monaco rule, is followed in 90% of cases. However, there are notable exceptions in which a severe DMD pathology was observed with an in-frame mutation [11]. One plausible explanation is that those “in-frame” mutations can impair normal pre-mRNA processing and suppress subsequent protein translation. Another theory suggests that those in-frame mutations actually land on essential domains of dystrophin, thereby disrupting protein function and causing severe DMD symptoms [9,11].

**Figure 1:**
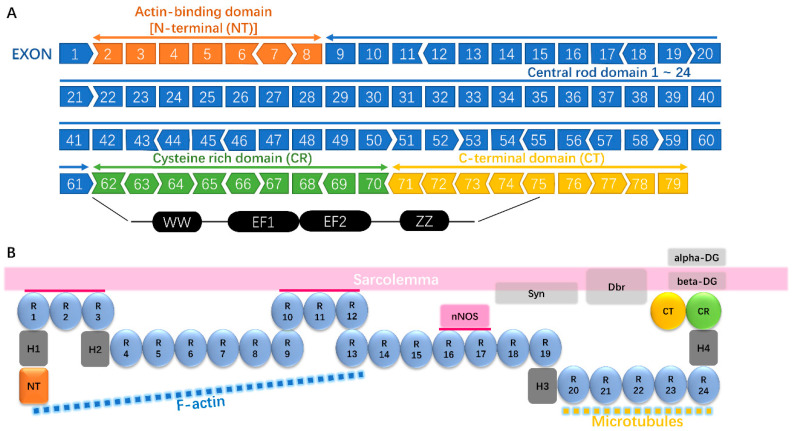
*Dystrophin* gene with exon mutation spots and their corresponding domains. (**A**) The structure of *dystrophin* gene. *Dystrophin* gene contains 79 exons. *N*-terminal domain (NT): exon 2–8; Central rod domain: exon 9–61; Cysteine-rich domain (CR): exon 64–70; C-terminal domain (CT): exon 71–79. The arrow shape of the adjacent exons shows open reading frame (ORF) compatibility. The CR and CT domains comprise the WW domain, EF hand and ZZ domains. (**B**) The schematic of dystrophin protein structure and dystrophin-sarcolemma interaction. In skeletal muscle, central rod domain 1–3 and 10–12, CR, CT binds to the sarcolemma, termed membrane binding domains (MBDs). In cardiac muscle, R10–12 do not bind to the sarcolemma. The *N*-terminal domain contains the primary actin binding domain which connects F-actin. The CR and first half of the CT bind to transmembrane β-dystroglycan. CT contains the dystrobrevin- and syntrophin-binding sites, which bind to the two transmembrane proteins on sarcolemma. The NT, CR, and CT are considered essential for dystrophin function. R: rod domain. H: hinge.

In general, a severe DMD phenotype can be induced by the following mutation patterns [8,9,10]: out-of-frame large mutations such as deletion of exon 45 and duplication of exon 2; in-frame mutations that disrupt or remove critical domains, including the cysteine-rich domain (exon 64–69), all four actin-binding sites (exon 2–8, 42–46), and a great proportion of the central rod domain (exon 9–61); widely distributed nonsense mutations; frame-shift small mutations like c.372delG in exon 6; and splice-site mutations resulting in inclusion of pseudoexons that contain premature termination codons. Those mutations lead to severe DMD pathologies as they produce prematurely truncated, unfunctional dystrophin protein, which will be quickly degraded [12]. Interestingly, Heald et al. [13] reported a case in which the patient with in-frame deletion of exon 3 to 9, which compromise most of the *N*-terminal actin-binding domain of the *dystrophin* gene, showed no symptoms and was diagnosed as BMD at age 67. This is likely due to the preservation of ABD2 in the rod domain (R11–15) to perform the binding function [14,15]. A schematic diagram of the *dystrophin* gene with exon mutation spots and the corresponding domains is presented in Figure 1. 

Notably, DMD mutation frequencies vary in terms of geography and race [8]. Genetic analysis of DMD patients showed that large deletions are the most common type of mutations worldwide (64% in Oceania, 66% in Europe, 70% in Americas, 72% in Asia, and 88% in Africa). Population-based cohort studies (Chinese [16], Spanish [17], and Italian [18]) also manifested racial characteristics of DMD mutation types.

### 2.3. Diagnosis Techniques Targeting Mutated Exons

Precise characterization of the DMD mutations and accurate diagnosis tools are important for genetic counseling and personalized medicine. Multiple PCR, MLPA (multiplex ligation-dependent probe amplification), and NGS (next-generation sequencing) are “the standard techniques” for DMD/BMD diagnosis. They are often applied in combination due to their unique strengths and weaknesses when used alone [19]. Multiple PCR was generally adopted as the first-step diagnosis maneuver for identification of large deletions. It is still widely used in some laboratories as it is cost-efficient and easy to operate. However, multiple PCR is unable to detect duplications, define deletion boundaries, or determine reading frame disruption [19]. MLPA, on the other hand, can identify both deletions and duplications by screening all the 79 exons simultaneously [20], yet it cannot detect small mutations. False positive results are sometimes obtained from MLPA analysis owing to contamination or sequence variations at the probe-binding sites [21]. Furthermore, when negative results are obtained from PCR and MLPA tests in highly suspected cases, judging from clinical manifestations, NGS can be applied to uncover small mutations including point mutations, small deletions, and duplications or insertions in exons, promoters, or known intronic mutations. As NGS may miss deep intronic or splice motif mutations that compromise normal splicing, hybrid minigene splicing assay, which can assess aberrant splicing of the *DMD* gene, becomes a reliable complimentary option. Hybrid minigene splicing assay allows relatively fast functional tests to recognize splice site mutations that cannot be identified by the three methods mentioned above [22]. Finally, if genetic testing is inconclusive, immunohistochemistry or Western blot analyses are definitive tests to confirm dystrophin expression in the muscle biopsies [23].

It has been estimated that 30–40% of DMD mutations are de novo [8,16,24]. Every year, new mutations appear, such as complex genomic rearrangements [25], deep intronic mutations that alter splicing patterns [26], etc. These novel, low-frequency mutations in non-hot spot sites demand more accurate diagnostic techniques for individualized management of DMD.

## 3. Stem Cell-Related DMD Pathogenesis

Although primarily classified as a muscle disorder, DMD is also considered as a stem cell disease [6]. DMD mutations result in progressive and irreversible muscle loss in patients. The only hope to “reverse” the condition lies in the activation of genomically intact muscle stem cells to regenerate muscle fibers. Thus, SC-related pathogenesis and therapies have attracted increasing attention in the recent years.

**Figure 2:**
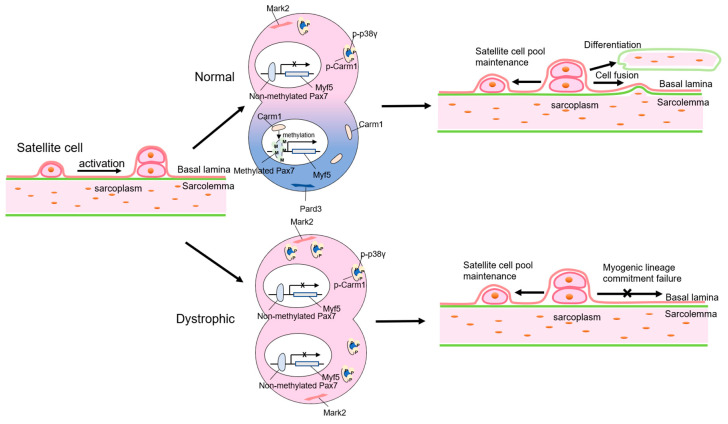
How intrinsic *DMD* gene deficit affects satellite cells (SC) activity and function. Normal SC performs asymmetric division when activated, while dystrophic SC fails to complete myogenic lineage commitment. Cell polarity regulator *Mark2* is repressed in dystrophic SCs, resulting in the absence of Pard3 protein in the apical position. Carm1 in dystrophic SCs is inactivated by p38γ, leading to *Pax7* methylation deficiency and subsequent inhibition of *Myf5* expression. Wild-type myogenic cells fuse with existing muscle fibers or differentiate to form new muscle fibers, whereas dystrophic satellite cells lose their differentiation capacity.

DMD mutations abolish dystrophin expression in SCs [6], which directly affects cell polarity and mitosis. Figure 2 summarizes the underlying mechanisms by which dystrophin controls SC activity and function. The Ser/Thr kinase *Mark2* pathway, a key factor regulating cell polarity, was dramatically downregulated in dystrophic SCs, causing the nonapical localization of Pard3 protein [27]. In addition, the uniformity of Mark2 protein in dystrophic SCs inactivated *Carm1* and subsequently reduced *Pax7* methylation [28]. As such, SCs lacking functional dystrophin undergo aberrant asymmetric division with impaired myogenic differentiation [29]. Moreover, dystrophin deficiency-associated mitotic defects such as centrosome amplification, spindle orientation mistakes, and prolonged cell cycle also exacerbate abnormal asymmetric division [27]. In a recent study, upon dystrophin restoration by CRISPR/Cas9, SCs became more resistant to endoplasmic reticulum stress and oxidative stress and had improved cell survival, proliferation, and differentiation [6]. Taken together, loss of dystrophin not only affects differentiated myofibers but also modulates stem cell viability and functions.

Several signaling pathways have been shown to control SC quiescence, senescence, and cell fate in dystrophic mice. Mu et al. reported increased Notch expression in dystrophin/utrophin double knockout mice [30]. Over-activation of *Notch* signaling contributed to SC senescence, whereas *Notch* inhibition improved cell function, implicating the clinical value of *Notch* inhibitor DAPT [30]. *Wnt-TGFβ2* pathway controls satellite cell fate, and dystrophic skeletal muscles exhibited increased level of Wnt3a and TGFβ2 [31]. Consistently, dystrophin-deficient SCs favor fibrogenic differentiation while impeding myogenic development [31]. Losartan (*TGFβ2* inhibitor)-treated mdx mice (a commonly used mouse model of DMD) had high levels of myogenic factors with decreased expression of fibrogenic genes [31], opening up a potential treatment for DMD patients using the US Food and Drug Administration (FDA) approved drug—losartan.

One of the key features of muscular dystrophy is chronic and dysregulated inflammation [32]. Upregulated inflammatory cytokines like TGF-α [33] and IL1-β [34] in dystrophic muscles activate *NF-κB*, a well-known proinflammatory transcription factor, resulting in repression of *MyoD* expression [35]. On the other hand, blockage of *NF-κB* promotes stem cell differentiation, and systemic administration of *NF-κB* inhibitor (sodium salicylate) enhances muscle regeneration in aged wild-type mice [36]. Moreover, transient depletion of macrophages in the mdx mice accelerates the adipogenic conversion of myogenic SCs through *IL-10* signaling [37], revealing the central role of macrophages in maintaining a “healthy” SC niche.

Hyperactivity of chicken ovalbumin upstream promoter–transcription factor II (*COUP-TFII*) was identified in both DMD patients and the mdx mice. Following these observations, Xie et al. [38] created transgenic mice in which *COUP-TF II* was ectopically expressed in muscle stem cells. They reported that SC dysfunction provoked Duchenne-like dystrophy in the wild-type mice and exacerbated degenerative myopathies in dystrophic animals, supporting a critical role of SCs in DMD pathogenesis [38]. Next, the authors determined that SC proliferation and differentiation, in particular myoblast fusion, were significantly compromised upon *COUP-TF II* activation. Furthermore, this group demonstrated the therapeutic potential of *COUP-TF II* antagonist by generating SC-specific *COUP-TF II*-deficient mdx mice, in which the progressive dystrophic symptoms were mitigated following *COUP-TF II* ablation. Recently, Petrany et al. [39] reported that SC-specific depletion of fusogenic protein Myomaker aggravated the dystrophic pathology in the mdx mice, confirming the effects of myoblast fusion in DMD pathophysiology.

Lu et al. [40] observed a rapid exhaustion of SC pool in dystrophic animals, presumably due to repeated cycles of myofiber degeneration–regeneration. However, as mentioned above, various mechanisms, including prolonged SC division, decreased SC self-renewal and alteration of stem cell fate, all contribute to stem cell exhaustion in the DMD scenario. Together, these studies uncovered the central effects of muscle stem cell in DMD progression, highlighting a potential therapeutic opportunity for managing degenerative muscle diseases.

## 4. Therapeutic Strategies for DMD

Based on the mutation types, several strategies targeting dystrophin restoration have been proposed years ago and are currently under investigation. Stop-codon read-through utilizes certain drugs or chemicals to selectively induce ribosomal read-through of premature stop codons. Theoretically, it is applicable to all nonsense mutations, which represent up to 10% of all DMD cases [8]. Exon skipping, an approach targeting affected exons with predesigned antisense oligonucleotides (AONs) to produce a shorter but working version of dystrophin, can treat 55% of DMD patients [8]. Vector-mediated gene therapy delivers functional *DMD* gene to cells lacking dystrophin protein [41]. Another emerging strategy is CRISPR/Cas9-mediated gene editing. Recently, several studies have combined CRISPR/Cas9 gene editing with cell therapy to achieve more prominent and permanent therapeutic effects [7,42]. Moreover, transplantation of myogenic cells capable of producing functional dystrophin protein has also been explored as an alternative approach [43].

### 4.1. Read-Through Therapy

Nonsense mutations are detected in approximately 10% DMD patients [8], and it usually produces a nonfunctional dystrophin protein. Additionally, the mRNA generated from nonsense mutations is destabilized by nonsense-mediated mRNA decay [44]. Read-through therapies utilize small molecules to interact with ribosome, which leads to insertion of an alternative amino acids at the point of premature termination codon to allow translational read through, so that a relatively functional dystrophin protein could be generated. Several medications have been developed based on this principle [45].

#### 4.1.1. Antibiotics and Synthetic Analogues that Mediate Stop-Codon Read-Through

Gentamicin, an aminoglycoside antibiotic, was proved to have read through ability. In a study conducted in 1999, gentamicin administration restored dystrophin expression in the mdx mice (harboring a nonsense mutation in exon 23) to almost 20% of the normal level [46].

However, the overall effectiveness of gentamicin is unclear and can be quite limited as clinical trials on humans showed mixed results. One study reported that among 12 patients (aged from 5 to 15) who received gentamicin for 6 months, 6 patients exhibited significantly increased dystrophin levels in their muscle tissues [45]. However, full-length dystrophin was not detected in another clinical trial involving 4 patients [47]. Despite some positive outcomes, inconsistent results of gentamicin treatment were observed in animal models and clinical trials [48,49]. Gentamicin is a mixture of major and minor aminoglycoside components, and read-through capacity of each component varies. Furthermore, there are significant differences in the pharmacokinetic characteristics among different gentamicin components. Together, these may explain the variable curing effects of gentamicin in the previous studies [48]. Additionally, the side effects of gentamicin put patients at risk for nephrotoxicity, neurotoxicity, cytotoxicity, and bacterial resistance [49,50].

To reduce the adverse effects of gentamicin, another antibiotic negamycin and its synthetic analogues were developed. Two negamycin analogues developed in 2017, 3-epi-deoxynegamycin and leucyl-3-epi-deoxynegamycin, could increase read through efficiency and promote dystrophin production without an obvious antimicrobial effect [50]. Another negamycin analogue (TCP-1109(13x) synthesized in 2019, exhibited a fourfold increase in read-through capacity compared with its competing analogue TCP-112(7) [51]. Together, these findings support a promising potential of negamycin analogues in the treatment of DMD. Further studies are in progress towards their clinical application.

#### 4.1.2. Ataluren-Mediated Stop-Codon Read-Through

Ataluren (3-(5-(2-fluorophenyl)-(1,2,4]oxa-diazol-3-yl)-benzoic acid), a compound formerly termed as PTC124, also suppresses nonsense mutations with low toxicity [52]. In a phase III trial (NCT01826487), the 6-min walking distance (6MWD) was not significantly improved between treated patients and the placebo group. Interestingly, a dramatic improvement was recorded in the prespecified subgroup of patients with a baseline 6WMD of 300–400 m. Ataluren is well tolerated. However, the efficacy of ataluren has been inconclusive as no data was provided to demonstrate dystrophin restoration in ataluren-treated patients. In 2014, Translarna (ataluren) was licensed in the European Economic Area for the treatment of nonsense DMD mutations in ambulatory patients aged 2 years and older. In April 2019, Translarna was granted marketing approval from the Brazilian National Health Surveillance Agency for the treatment of ambulatory DMD children of 5 years and older (https://ir.ptcbio.com/news-releases/news-release-details/translarnatm-ataluren-first-therapy-approved-brazil-duchenne). In contrast, ataluren has been rejected by FDA twice. The latest study aiming to assess the safety and pharmacokinetics of ataluren in DMD patients (6–24 months) was posted on 7 April 2020 (NCT04336826). It is estimated to complete in 2023.

### 4.2. AON-Mediated Exon Skipping Therapy

Internally truncated dystrophin protein found in BMD is partially functional and provides the basis for exon skipping therapy [53]. Exon skipping is achieved by administration of 20–30 bp long antisense oligonucleotides. AONs specifically hybridize to splice motifs essential for pre-mRNA processing and mask the splicing signals on the RNA, leading to the exclusion of both the intron and its adjacent exon. Thus, an in-frame mRNA without the targeted exon is generated, and a truncated but still partially functional dystrophin can be translated. The latest and ongoing clinical trials of exon skipping drugs are summarized in Table 1.

Exon skipping AON is mutation specific and multiple drugs are needed to cover a large group of patients. According to a large dataset published in 2015, DMD mutations mainly occur in hot spot regions [8]. The top 5 mutated exons found in DMD patients were exon 51 (14% of total mutations/21% of deletions), exon 53 (10%/15%), exon 45 (9%/13%), exon 44 (7%/11%), and exon 43 (7%/11%) [8]. As such, drugs targeting exon 51 (eteplirsen, drisapersen, etc.), exon 53 (golodirsen, viltolarsen), and exon 45 (casimersen) are tested in clinical trials. Among them, eteplirsen, golodirsen, and viltolarsen are now available for DMD patients [54].

#### 4.2.1. Phosphorodiamidate Morpholino Oligomer (PMO) Modification

PMOs are synthetic DNA analogs containing a backbone of six-sided morpholine rings that are connected to each other by phosphorodiamidate linkage [55]. PMOs are neutrally charged, which provide better tolerability by reducing off-target effects and immune responses [56]. The advantages of PMO have led to the consecutive development of several drugs: eteplirsen and golodirsen have received conditional approval from FDA for skipping exon 51 and 53, respectively. Viltolarsen, which targets exon 53, was approved in Japan. Casimersen (skipping exon 45) is currently under clinical trials. However, PMOs have certain limitations. First, the pharmacokinetics of PMOs showed limited cellular uptake, rapid clearance from systemic circulation, and short duration of the exon-skipping effect. Therefore, high and repeated doses is necessary for clinical usage [57]. Second, animal studies conducted in mice and dogs showed poor penetration of PMOs in cardiomyocytes, leading to limited exon-skipping efficacy in heart muscles [58,59,60].

In April 2016, FDA Advisory Committee rejected accelerated approval for eteplirsen with a split decision of 7:6. Later, eteplirsen received FDA conditional approval as it increased dystrophin production in DMD patients from 0.16% to 0.44% over a 48-week therapy and from 0.28% to 0.93% after 188 weeks of treatment [61]. In another study involving 12 patients, there was a statistically significant improvement on 6MWD test following 36-month treatment [62]. However, as the small sample study was not very compelling, the same committee voted 7:3 against its full approval. As such, Sarepta Therapeutics (Cambridge, MA, USA) was required to provide confirmatory data by 2021 for the final approval of eteplirsen. 

In December 2019, intravenous golodirsen was approved in the United States to treat DMD patients [63]. In the 168-week phase II study involving 25 patients, all participants showed exon 53 skipping response at week 48, along with an increased dystrophin expression (from 0.095% to 1.109%) [64]. Although animal studies suggested golodirsen may cause kidney dysfunction, renal toxicity was not observed in treated patients. However, Sarepta Therapeutics needs to report disease progression in those patients by 2024 [65].

Like golodirsen, viltolarsen also targets exon 53. However, viltolarsen is a 21-mer, whereas golodirsen is a 25-mer oligonucleotide. Thereby, for the same dose, the amount of AON in viltolarsen would be 20% more than that in golodirsen [66]. Viltolarsen has been tested in Japan (20, 40, and 80 mg/kg per week via intravenous infusion) and in the United States (40 and 80 mg/kg per week via intravenous infusion). After 24 weeks of treatment, there was a 5.8% increase of dystrophin protein from baseline in muscle biopsies. As such, FDA has accepted the New Drug Application (NDA) under the priority review for viltolarsen. In March 2020, intravenous viltolarsen got its first approval in Japan for treating DMD patients with mutations amenable to exon 53 skipping. Currently, the clinical trials of viltolarsen are undertaken in the United States and Canada [67].

Casimersen, an oligonucleotide that skips exon 45 [54,65], is also in clinical development. In 2015, a phase III trial with 99 patients was conducted to evaluate the efficacy of SRP-4045 (casimersen) and SRP-4053 (golodirsen) for 48, 96, and 144 weeks of treatment (NCT02500381). Primary results updated in 2018 showed improvements in the 6MWD test at week 96. Another open-labeled extension trial enrolling 260 patients was initiated in 2018 to assess the safety and tolerability of long-term (144 weeks) casimersen or golodirsen medications (NCT03532542). Recently, Sarepta Therapeutics has submitted an NDA to FDA, seeking accelerated approval for casimersen. The submission includes data from a global, randomized, double-blind, placebo-controlled phase III study in which a statistically significant increase of dystrophin protein was identified in treated patients as compared to the placebo group and the baseline. (https://investorrelations.sarepta.com/news-releases/news-release-details/sarepta-therapeutics- completes-submission-new-drug-application-0).

#### 4.2.2. 2′-O-Methyl-Phosphorothioate (2′OMePS) Modification

2′OMePS is an AON in which the phosphorothioate backbone is modified by methylation of the 2′ position in the ribose ring. Replacing the nonbridging oxygen in the phosphate group with a sulfur atom prevents its immediate degradation by endonucleases [55]. However, delivery to the target site and adverse effects related to off-target exposure remain challenging. Drisapersen is a designed 2′OMePS compound that targets exon 51. In two phase II trials (NCT01153932 and NCT01462292) [68], patients treated with drisapersen showed improvements in 6MWD tests. Although muscle function, pharmacokinetics, and dystrophin level were evaluated in this trial, treatment-related increase of dystrophin protein was not observed in immunofluorescence (*n* = 34) or Western blot (*n* = 11) analyses, making the results inconclusive. Additionally, a large-scale phase III clinical trial with 186 participants (NCT01254019) presented controversial and disappointing results on delaying ambulation [2,69]. 

#### 4.2.3. Peptide-Conjugated PMO (PPMO)

To enhance PMO penetration of cell membrane, peptide-conjugated morpholino oligomer (PPMO) has been developed. PPMOs attach a short arginine-rich cell-penetrating peptide fragment (peptide nucleic acid (PNA) internalization peptides, Pip) to PMO at either the 5′ or 3′ end by direct chemical conjugation [55]. Compared with PMO, PPMO is more stable in the circulation system and can be efficiently delivered to cells [44]. Thus, PPMO may solve the low delivery efficiency of PMO, especially into cardiac muscles. A series of Pips have been developed, among which Pip5 and Pip6 showed efficient targeting activity to cardiac myofibers. However, PPMO displays higher toxicity than PMO because it is positively charged. When administered to animals at a high dose, PPMO induced lethargy, weight loss, and renal toxicity [70], which is likely due to arginine residuals. Newly developed Pips (series 7,8,9) reduced the number of arginine residues (from 10 to between 6 to 9), which significantly decreased toxicity but also lowered splice-switching activity [71]. Nevertheless, PPMO is the most advanced and promising AON with the potential to overcome major limitations of other AONs.

Sarepta Therapeutics has redesigned the eteplirsen with PPMO that contains two arginine-rich domains separated by a central short hydrophobic core, named SRP-5051. Animals treated with SRP-5051 exhibited improved tissue uptake and increased dystrophin restoration in both skeletal and cardiac muscles [72]. At present, Sarepta Therapeutics is conducting additional phase II and phase I/II open-label extension studies to investigate the safety and tolerability of SRP-5051 (NCT04004065 and NCT03675126).

#### 4.2.4. Stereopure AON

PMO and PPMO are synthesized AONs with modification molecules randomly located at each linkage of nucleotides, adopting either Sp or Rp position. Thus, more than 500,000 (2^19^ = 524,288) permutations could appear in a 19-nucleotide sequence, which can cause off-target effects [68]. Stereopure AON represents a more homogeneous set of AONs generated in a stereoselective manner. This stereochemistry enables optimization of critical constructs into one defined and consistent profile, which is safer and more effective. Wave Therapeutics recently developed suvodirsen (WVE20201) that increased natural dystrophin production in vitro, improved oligonucleotide uptake in the nucleus, and was rapidly cleared from liver and kidney when administered in mice. However, suvodirsen treatment did not promote dystrophin restoration in DMD patients after 12 or 22 weeks of therapy. Therefore, Wave Therapeutics has permanently suspended all their DMD trials. Whether this reflects a fundamental problem of stereopure compounds, or the inherent difficulty of the delivery needs further investigation.

#### 4.2.5. Efficacy and Safety of AON-Mediated Exon Skipping

Drug effectiveness and safety are essential for the development of AON-mediated exon skipping. One strategy is to develop more efficient AON modifications such as PPMO, arginine-rich peptide PMO, 2OMePS, stereopure AON, etc. A study conducted in 2019 found that locked nucleic acid/2′-O-methyl mixmers increased exon skipping efficiency [73]. Another approach is to enhance target-tissue uptake using adjunctive components like glycine [74]. One important consideration in DMD therapy is the skipping efficacy in the cardiac muscles given that cardiomyopathy is the leading cause of death among DMD patients. Unfortunately, AON shows negligible effects in dystrophin restoration in cardiac muscles compared with skeletal muscles [75]. For instance, PMO is usually trapped in endosomes in cardiomyocytes with limited permeability of membrane barriers in cardiac muscles [58,76]. Several approaches have been employed to enhance the effectiveness of antisense drugs such as the usage of tricyclo-DNA, peptide-conjugated PMO (B peptides, PNA/PMO internalization peptides, and phage peptides), octaguanidine morpholino, ultrasound and microbubbles, and nanoparticles [76]. Among these drugs, only PPMO treatment resulted in considerable expression of dystrophin in the cardiomyocytes in dystrophic animals [77], making it a promising AON of drug development. 

Another concern is drug safety. Although PPMO exerts efficient cellular uptake, the toxicity associated with these arginine-rich peptides remains challenging [78]. For instance, PPMO has been shown to cause lethargy in mice [70], and studies in nonhuman primates confirmed the harmful effects of the drug [71]. The underlying mechanisms are not clear but likely related to treatment duration, administration frequency, dosage, the exon targeted, the cationic nature of peptides and immune response, etc. [55]. Currently, reducing the toxicity while preserving the activity of PPMO is a hot research topic in exon skipping. Finally, for all the AONs, drug toxicity is generally dose dependent, thus determining the maximum tolerated dosage is critical in drug development.

### 4.3. Vector-Mediated Gene Therapy

Transfer of normal *DMD* gene into dystrophic muscles has been a logical therapy upon its first proposal. However, it is challenging because of the enormous size of the *dystrophin* gene and the widespread distribution of muscles [79]. Adeno-associated virus (AAV)-mediated mini-/microdystrophin transfer [79] and artificial chromosome-mediated dystrophin delivery [80] are the major approaches in vector-mediated gene therapy.

#### 4.3.1. AAV-Mediated Mini-/Microdystrophin Transfer

*Dystrophin* gene is 2.3 mb in length with 79 exons [7]. Due to the difficulty in virus packaging with full-length *dystrophin* gene, the mini-/microdystrophin therapies were developed. The microdystrophin genes are about 4 kb length without the C-terminal domain [81]. The recombinant AAV virus was generated by Hermonat et al. in 1984 [82], and *dystrophin* gene transfer utilizing AAV has been investigated since then [79]. In a recent study, rAAV2/8 was employed to deliver canine microdystrophin gene to dystrophic dogs. Upon treatment, sustained dystrophin protein was detected in averagely 50% of limb skeletal muscles, whereas less than 0.5% of the cardiomyocytes manifested microdystrophin expression was observed. Western blot showed 60–80% microdystrophin restoration compared to wild-type cells, and no toxic effects or immune responses were observed [83]. However, this type of locoregional administration has very limited effect in whole body dystrophin restoration and muscle function recovery.

While microdystrophin cannot fully restore the function of WT dystrophin without C-terminal domain, the 6 kb minidystrophin gene contains all functional domains and displays better bioactivity compared to microdystrophin [84]. However, the restricted AAV packing size (4.7 kb) limits its delivery capacity of minidystrophin gene. Recent studies on trans-splicing AAV system can double or even triple the maximum packing size of AAV [85]. Koo et al. has successfully developed a triple-AAV trans-splicing vector system in which three independent AAV vectors carrying sequential parts of the *DMD* gene can be cojoined to generate full-length dystrophin protein through trans-splicing events [85]. Expression of full-length dystrophin protein was achieved in 4% of total skeletal muscles following intramuscular injection [85].

Dosage-dependent immune responses elicited by AAV-mediated gene delivery are the major safety and tolerability concerns for its clinical usage, especially in high dose delivery. AAV gene therapy triggers immune response to both AAV capsids and the transgenes delivered. In a study conducted by Kornegay et al., high dose (1.5 × 10^14^ vg/kg) of AAV administration via intravascular injection induced inflammatory myopathy in two dogs. No CD4^+^ or CD8^+^ T cells were observed in microdystrophin^+^ muscle fibers, indicating the occurrence of innate immune response rather than cell-mediated immune response [86]. Martino et al. reported transient induction of pro-inflammatory cytokines after intravascular delivery of AAV2 and AAV8 capsids and showed that higher delivery dosage would lead to stronger innate immune responses [87]. Meanwhile, immune response to mini-/microdystrophin were also demonstrated. The first minidystrophin clinical trial was unsuccessful as patients failed to express the synthetic minidystrophin gene, presumably due to the induction of immune responses against the minidystrophin. The specific T cell cluster against minidystrophin were detected even when synthesized protein was not expressed [88]. Recently, Stedman’s group provided a possible solution [89]. Utrophin, a structural paralogue of dystrophin, can substitute the function of dystrophin. Stedman’s team designed a miniaturized utrophin (μUtro) which was highly functional and nonimmunogenic. As reported, μUtro with the presence of dystrophin-glycoprotein complex (DGC) were widely detected in muscles and myonecrosis were suppressed upon AAV-μUtro injection [89]. More importantly, there is no evidence of cell-mediated immune response against μUtro, supporting a view that μUtro can be used to treat DMD with a favorable immunologic profile [89].

As summarized in Table 2, several clinical trials on AAV-mediated dystrophin transfer have been carried out globally over the past few decades. Still, challenges remain regarding limited transfection efficiency, optimizing administration method, and managing immune responses. Factors including patient age, AAV cassette promoter, AAV serotypes, dosage, and delivery methods should all be carefully assessed. Most of the participants in the above listed trials are children and adolescents, except two trials (NCT03333590 and NCT02376816) in which adults were recruited as well. One complete trial (NCT00428935) used ubiquitous promoter (CMV) as AAV promoter, whereas other five trials chose muscle specific promoters, such as CK8 (NCT03368742), MHCK7 (NCT03375164), and MCK (NCT03362502, NCT03333590, and NCT02376816). Several AAV serotypes including AAV2.5 (NCT00428935), AAV9 (NCT03368742 and NCT03362502), and AAVrh74 (NCT03375164, NCT03333590, and NCT02376816) have been tested. Multigradient doses were applied in most trials except the one conducted by Sarepta Therapeutics (NCT03375164). Virus was injected intramuscularly (NCT00428935 and NCT02376816) or intravenously (systemic infusion, NCT03368742 and NCT03362502; limb-specific infusion, NCT03375164 and NCT03333590). 

Phase I trial conducted by Nationwide Children’s Hospital examined the safety and tolerability of empty AAV capsids, and primary outcomes indicated that AAV2.5 vector was safe and well tolerated [90]. However, one trial with secondary outcomes showed no dystrophin expression, whereas specific T cell clusters against translated dystrophin protein were detected, implicating the potential safety concerns related to T-cell immunity targeting self and nonself-dystrophin epitopes [88]. Based on the limited clinical data, it is difficult to determine the optimal administration dosage and the therapeutic window. Adeno-associated viruses are attractive gene therapy vectors due to their relatively low toxicity, rare integration into the host genome, and the ability to persist for months to years. However, because its episomal expression may decrease after cell division, thus readministration of the AAV vectors may still be required [91,92]. For example, the abovementioned study carried by Le Guiner et al. suggests that systemic administration of microdystrophin can lead to a persistent gene expression for 2 years [83], indicating the stability of systemic AAV delivery and the necessity of readministration. Given that DMD is a progressive disease and T cell-mediated immunity requires long-term exposure to AAV, choosing a well-tolerated AAV vector and low-dose administration at young ages might reduce AAV-triggered immune response.

Unfortunately, in a recent clinical trial of gene transfer in X-Linked myotubular myopathy (NCT03199469), two patients who received high dose of AAV8 (3 × 10^14^ vg/kg) via intravenous administration, showed serious adverse events and died because of progressive liver dysfunction (https://www.joshuafrase.org/get-involved/recensus-study.php). Preliminary analyses suggest an involvement of immune response triggered by AAV capsids or the carried genes. Notably, those patients share similar features including older age, heavier weight, and pre-existing hepatobiliary disease. Therefore, the dosage, readministration frequency, patient age, and pre-existing diseases should be prudently evaluated before and during clinical application.

#### 4.3.2. Artificial Chromosome-Mediated Dystrophin Transfer

Human artificial chromosome (HAC), which is derived from native chromosome by “top-down” engineering or “bottom-up” de novo synthesis, holds the potential to deliver full-length *DMD* gene into patients [80]. HAC can replicate during mitosis as an extra genomic copy. Kazuki et al. generated dystrophin-HAC in iPSCs derived from DMD patients [93]. These iPSCs differentiated into mesoangioblast-like myogenic progenitors with restored dystrophin expression. However, whether this iPSC-HAC can deliver the entire *dystrophin* gene into dystrophic muscles is unknown. On the other hand, the possible immune rejections triggered by exogenous chromosomes must be taken into consideration [80]. Ethical issues of using human iPSCs as the delivery vector need to be evaluated as well [94].

### 4.4. CRISPR/Cas9-Mediated Gene Editing 

To obtain permanent dystrophin expression in DMD patients, gene editing, particularly CRISPR/Cas9-based gene editing, has been adopted to correct genomic deficits. CRISPR/Cas9 system consists of the endonuclease Cas9 and a single-guide RNA (sgRNA) [95]. The Cas9 endonuclease produce DNA double-strand break at the editing sequence targeted by sgRNA. Subsequent nonhomologous end-joining (NHEJ) leads to exon-skipping while homology-directed repair (HDR) could replace DMD mutations with correct sequences and generates normal dystrophin protein [95]. Two types of Cas9 endonucleases have been used for CRISPR gene editing, *Staphylococcus aureus* Cas9 (SaCas9) and *Streptococcus pyogenes* Cas9 (SpCas9). SaCas9 is about 1 kb smaller than SpCas9, which allows it to be packed more efficiently into AAVs, leaving additional space for crRNA and tracrRNA [96,97]. The smaller size also makes it possible to build SaCas9 and gRNA into a single plasmid, the so-called all-in-one vector. Moreover, the longer PAM sequence when using SaCas9 leads to more accurate editing with decreased off-target effects. Hence, SaCas9 is more suitable for in vivo gene editing as compared to SpCas9.

#### 4.4.1. Ex Vivo CRISPR/Cas9 Gene Editing

Young et al. applied CRISPR/Cas9 technique in iPSCs derived from DMD patients [98] in which the gRNA targeted intron 44 and 55 to flank exon 45–55, which is the most frequently mutated region (60%) in DMD patients. Those iPSC-derived skeletal myocytes expressed dystrophin^Δ45–55^. When transplanted into the mdx mice, corrected cells mitigated muscle breakdown as shown by decreased creatine kinase level. Moreover, the expression of β-dystroglycan, a member of DGC complex, was also restored [98]. Exon 44 deletion causes splicing of exon 43 to exon 45 and results in premature termination codon. A study conducted by Min et al. corrected an exon 44 deletion mutation and restored dystrophin expression in iPSC-derived cardiomyocytes [99]. 

Another strategy worth mentioning is CRISPR gene editing in muscle stem cells. Zhu et al. [100] carried out CRISPR-mediated HDR in dystrophic SCs and restored dystrophin expression. Although limb muscles from dystrophic mice exhibited dystrophin restoration upon transplantation of edited SCs, there was no measurement of muscle function in this study.

#### 4.4.2. In Vivo CRISPR/Cas9 Gene Editing

In vivo CRISPR/Cas9 strategies have also been studied in DMD therapy, most of which choose NHEJ to achieve exon-skipping and subsequent dystrophin restoration [95]. Majority of the flanking regions are chosen within exon 45–55, while other regions like exon 21–23 have also been tested [95,99,101]. Administration routes include intramuscular or intravenous injection, pronuclear packaged nanoparticle microinjection and intramuscular electroporation of AAV or adenovirus (ADV) [95]. Xu et al. induced in-frame deletion of exon 21–23 in mice by ADV containing two sgRNA and spCas9 endonuclease [101]. Upon intramuscular delivery of adenovirus, dystrophin restoration around injection region reached about 50% of wild-type level. Immunofluorescence staining confirmed the presence of DGC on sarcolemma [101]. Another research group designed a nanoparticle consisting of donor DNA, spCas9, and sgRNA. Two weeks after intramuscular injection into mdx mice, there was about 5% HDR-mediated genomic correction [102]. Edited myoblasts collected around injection site were induced to differentiate into myotube with 30–40% dystrophin restoration compared with wild-type cells [102]. Furthermore, Tabebordbar et al. performed in vivo gene editing of muscle stem cells via AAV-CRISPR system and demonstrated that those corrected SCs expressed dystrophin protein with increased differentiation capacity [103].

Taken together, gene editing technologies hold great potential for many inherited genetic disorders including muscular dystrophies. However, challenges remain in CRISPR-mediated gene editing. In general, NHEJ-mediated exon deletion show higher error rate as compared to HDR-mediated accurate genome correction. In contrast, the HDR editing efficiency is usually lower than that of NHEJ. As such, Chen et al. performed in vitro high-throughput compound screening and identified two small molecules, L755507 and Brefeldin A, which can enhance HDR genomic correction efficiency [104].

The CRISPR/Cas9 gene editing system relies on the recognition of specific sequence by sgRNAs. Due to the extreme complexity of the genome, sgRNA may be locally matched to other nontargeted sequences and result in off-target effects. To reduce the off-target exposure, researchers have optimized gRNA by adjusting its length and enhancing the stability [105,106]. Several spCas9 homologous enzymes including SaCas9, NmCas9, and StCas9 have been shown to increase targeting specificity [96,107,108]. Furthermore, Terao et al. constructed a fused endonuclease complex consisting of dCas9 and FokI endonuclease in which dCas9 only retained its sequence recognition ability. With the assistance of FokI endonuclease, gene editing can be achieved with low off-target effects [109].

### 4.5. Exogenous Cell Transplantation

Transplantation of muscle precursor cells (so-called myoblast) into mice was first proposed by Watt and Morgan [110]. The injected myoblasts fuse with host myofibers and express donor genes [110]. Subsequent experiments verified the presence of dystrophin in treated animals [111]. Clinical data demonstrated that muscles treated with myoblast transplantation possess a higher maximum voluntary force as compared to muscles in the contralateral side [112]. However, Western blot of patient biopsies indicated a low percentage (0–5%) of dystrophin expression, which can be partially explained by immune rejection or low survival of transplanted myoblasts [113]. Thereby, telomere elongation of the immortalized myoblasts has been applied to promote cell proliferation capacity [114]. However, a major concern of myoblast transplantation is the oncogenic potential of the immortalized cells. As such, conditional proliferation-dependent suicide agents like herpes virus thymidine kinase can be employed to reduce the risk of such adverse events [114].

Satellite cells hold specific advantages compared with myoblasts because of their self-renewal capacity and differentiation potential. When injected into mice, transplanted SCs formed stable engraftment in dystrophic muscles and established stem cell niche [115]. However, cultured SCs are heterogenous in their activation status and self-renewal ability, which may cause inaccurate “homing” of stem cells [116]. SC biomaterial culture medium and protein-mediated stem cell “homing” strategy [117] can maintain properties of stem cell including self-renewal and differentiation capacity. For instance, Gilbert et al. designed a PEG hydrogel system to mimic the natural SC niche [118]. Satellite cells grown in this 3D hydrogel system survive better and show increased engraftment and repopulation capacity when transplanted in vivo [118]. Hence, hydrogel-based SC culture represents a new strategy for continuous culture of stem cell and consecutive replenishment of SC pool in dystrophic patients.

Tissue engineering, such as cell-supportive scaffolds, can provide mechanical support and growth factors to promote satellite cell engraftment [119]. As myofibers are arranged in parallel order, the aligned nanofibrous mesh is conducive to muscle cell differentiation [120]. Ricotti et al. [120] evaluated the myogenic capacity of two myoblast cells, C2C12 and H9c2 cells, on an electrospun polyhydroxybutyrate (PHB) matrix with different fibrous orientation. Compared to polystyrene controls, flat and aligned nanofibrous PHB scaffolds efficiently drive myofiber formation of myoblasts [120]. Another study fabricated the nanofiber matrices by blending polycaprolactone (PCL) with polyaniline (PANi, a conducting polymer) [121]. Higher PANi concentration in the blended scaffolds provides a higher myogenic stimulus for C2C12 cells, supporting the synergic effects of electroactivity on myogenesis [121]. Moreover, chemical signals can be added to the scaffolds. Wnt3a incorporation into the chitosan–polycaprolactone matrix greatly enhanced the myogenic development of embryonic stem cells, demonstrating the superiority of combining biomaterial scaffolds with exogenous signaling molecules [122].

Human induced pluripotent stem cell (iPSC) is another tempting cell source for reconstructing dystrophic muscles. iPSCs cultured with cytokines and growth factors were committed to myogenic linage [123], while the forced expression of myogenic factors such as Pax7, Pax3, and MyoD also works [124]. The induced Pax3/Pax7^+^ PSCs can repair damaged muscles and reconstitute SC niche upon transplantation. Other myogenic cells with different origins like mesoangioblast were also explored for DMD treatment [125]. Isolated from embryonic dorsal aorta, mesoangioblast is the progenitor of pericytes that expresses both myogenic marker *MyoD* and endothelial marker *CD31*. Intravascular injection of mouse mesoangioblasts into dystrophic mice proved their in vivo migration and differentiation capacity [125].

### 4.6. Level of Functional Dystrophin Required for Clinical Efficacy

How much dystrophin restoration is required for clinical efficacy has always been an important issue in DMD therapy? In mice studies, Barton-Davis et al. [46] found that 20% of normal dystrophin could significantly reduce the force deficit associated with eccentric exercise compared with untreated mdx mice. Sharp et al. [126] reported a minimum of 20% of dystrophin-positive muscle fibers are necessary for meaningful improvement in muscle physiology. Another group [127] concluded that 15% of normal levels of dystrophin were sufficient to prevent the force drop in the tibialis anterior muscle of mdx mice. van Putten et al. [128] stated that “while even dystrophin levels below 15% can improve pathology and performance, more than 20% dystrophin restoration is needed to fully protect muscle fibers from exercise-induced damage.” On the other hand, there have been a few studies to address the effects of dystrophin level in canine models. Le Guiner et al. [129] showed the minimum threshold of “percentage of dystrophin-positive fibers” to exert therapeutic effects were 33% for structural improvements and 40% for strength measures. Another study conducted in dystrophic dog [130] concluded that proper muscle contractile function requests at least 40% dystrophin-positive fibers. 

In DMD patients, it has been shown that 30% dystrophin levels were sufficient to prevent muscular dystrophy [131]. Dystrophin abundance in healthy individuals differs from threefold to fivefold, thus the minimum dystrophin production required in dystrophic patients may also vary among individuals [132]. Notably, some patients with low levels of dystrophin still maintained a relatively normal muscle function [36]. In an early study, boys who had some dystrophin expression in their muscles lost independent mobility around age 10. In contrast, the mean age of losing mobility among DMD boys with no detectable dystrophin protein was 7.9 years [133]. In a recent case report, a 10-year-old boy with nonsense mutation in exon 42 expresses only 3.2% of dystrophin protein of the normal level. This boy has intermediate muscular dystrophy phenotypes, indicating that even low levels of dystrophin can mitigate skeletal muscle weakness [134]. A recent review [135] summarized multiple studies performed in mice, canine, and DMD patients and concluded that 20% of normal level of dystrophin is sufficient for therapeutic improvement. However, previous studies used different methods and indexes to measure dystrophin protein, making it difficult to compare results among different studies. Moreover, few studies have examined muscle functions after treatments, which renders the bridging between “dystrophin restoration level” and “clinical efficacy” more challenging. Future studies should standardize the measurement of dystrophin protein and assess dystrophin expression and muscle function at the same time.

## 5. Discussion and Future Direction

Duchenne muscular dystrophy is a progressive neuromuscular disorder. Mutations in the *DMD* gene abolish dystrophin expression in both mature muscle fibers and muscle stem cells, causing muscle wasting and satellite cell dysfunction. There have been continuous efforts to improve the diagnosis and to explore therapeutic approaches to treat this fatal disease. The most advanced therapy is AON-mediated exon skipping and several exon skipping drugs have been used in clinic application. Gene editing and cell-based therapies are novel strategies currently under investigation and modification. Here, we review these current and emerging treatments for DMD patients.

The exon skipping therapy has been proposed for decades and is available for patients’ use for several years. Two medications from Sarepta Therapeutics (eteplirsen and golodirsen) have received FDA accelerated approval and viltolarsen from Nippon Shinyaku Co. Ltd. has been approved in Japan. Global efforts to improve the effectiveness and safety of exon skipping AONs are undertaken. The emergence of PPMOs offers hope for overcoming the limitations of other AONs. The conjugation of arginine-rich peptides to PMO increases its serum stability. Numerous studies in animal models have demonstrated the advantages of PPMOs over PMOs, such as enhanced internalization into cells, increased potency at lower doses, efficient delivery to skeletal, respiratory, and cardiac muscles, and sustained dystrophin production in target tissues [44,55,70]. Exon skipping by PPMO is a revolutionary therapy that could be applicable to the majority of DMD patients. However, a major concern of PPMOs is their toxicity, which is not well understood. Hopefully, ongoing research will illustrate the beneficial mechanisms of PPMOs and facilitate the synthesis of novel PPMOs with maximum tissue uptake and minimum cytotoxicity. 

Recently, gene editing has emerged as a promising strategy to permanently correct DMD defects, thereby restoring dystrophin protein. Gene editing within myogenic stem cells, especially SCs, has the potential to maintain stem cell reservoir, restore dystrophin expression, and reconstitute muscle tissue in the long run [136]. Two approaches have been tested: in vitro gene editing of autologous stem cells followed by transplantation and in vivo gene editing, which affects all cell types exposed to editing components, including SCs. 

For in vitro gene editing of myogenic stem cells, CRISPR/Cas9 can be applied to both SCs and human iPSCs with editing efficiency ranging from 5% to 40% [98,99,100]. Although SCs have superior self-renewal capacity and differential potential, the scarce number makes it difficult for genome editing. On the other hand, patient-derived iPSCs provide a better cell source for gene manipulation [115,123]. Still, there are some technical barriers of this approach: (1) disturbances of stem cell niche and alterations of stem cell properties (loss of stemness) during in vitro culture and (2) challenges after transplantation such as stem cell migration and “homing” [116,137]. As mentioned earlier, to improve the efficacy of SC transplantation, bioengineering approaches such as hydrogel-cultured SCs have been explored [118,119]. 

In situ genome modification of stem cells detours from ex vivo gene editing, thereby preserving native regulatory interactions and stem cell properties [136]. However, there are technical difficulties such as low transduction efficiency of AAV into stem cells. For instance, a study carried out in 2014 found no detectable transduction of AAV vectors in SCs in vivo [138]. Another study showed restored dystrophin expression in mdx mice via transplantation of ex vivo edited autologous stem cells [100]. Contrary to previous findings, Goldstein et al. proved that AAVs are capable of transducing SC at efficiencies required for therapeutic gene editing upon systemic injection [136]. This result was obtained using a Cre/lox system coupled with a sensitive fluorescent reporter that is very efficient and sustainable. Several studies uncovered that rAAV serotypes 1, 7, 8, 9, and 10 were more efficient than the others, with rAAV9 being the best in mice [139,140]. Of course, for in vivo stem cell modification, issues such as AAV-associated immune responses and CRISPR-related off-target effects need to be addressed as well.

In conclusion, we propose directing more attention toward genomic editing in myogenic stem cells, especially SCs. We believe that gene therapy combined with cell transplantation and tissue engineering has the potential to lead to life-changing therapy for DMD, which is also applicable for other types of muscular dystrophies including Becker muscular dystrophy and distal muscular dystrophies.

## Figures and Tables

**Table 1:** Latest and ongoing clinical trials of exon skipping drugs.

Chemical	Drug	Other Name	Sponsor	Targeted Exon	Clinical Trial Number	Trial Phase	Start Date	Completion Date
PMO ^1^	Eteplirsen	AVI-4658, Exondys 51	Sarepta Therapeutics	Exon 51	NCT03218995	2	August 2017	March 2021
					NCT04179409	2	February 2020	September 2022
					NCT03992430	3	January 2020	October 2024
					NCT03985878	3	June 2019	February 2027
	Golodirsen	SRP4053	Sarepta Therapeutics	Exon 53	NCT04179409	2	February 2020	September 2022
					NCT02500381	3	September 2016	May 2023
					NCT03532542	3	2 August 2018	10 August 2026
	Casimersen	SRP4045	Sarepta Therapeutics	Exon 45	NCT04179409	2	February 2020	September 2022
					NCT03532542	3	2 August 2018	10 August 2026
	Viltolarsen	NCNP-01, NS-065	Nippon Shinyaku Co Ltd.	Exon 53	NCT03167255	2	July 2017	August 2021
					NCT04060199	3	April 2020	December 2024
2′-O-methyl PS	Drisapersen	PRO051, GSK2402968	BioMarin Pharmaceutical Inc.	Exon 51	NCT02636686	Extension	December 2015	January 2018
	DS-5141b		Daiichi Sankyo Co., Ltd.	Exon 45	NCT02667483	1, 2	October 2015	December 2020
PPMO ^2^	SRP-5051		Sarepta Therapeutics	Exon 51	NCT03675126	1, 2	December 2018	July 2024
					NCT04004065	2	June 2019	August 2021
Stereopure	Suvodirsen	WVE-210201	Wave Life Sciences Ltd.	Exon 51	NCT03907072	2, 3	September 2019	January 2020

^1^ PMO: Phosphorodiamidate Morpholino Oligomer; ^2^ PPMO: Peptide-Conjugated PMO.

**Table 2:** Clinical trials of Adeno-associated virus (AAV)-mediated microdystrophin transfer for Duchenne muscular dystrophy (DMD) therapy.

Sponsor	Nationwide Children’s Hospital	Solid Biosciences, LLC	Sarepta Therapeutics, Inc.	Pfizer	Kevin Flanigan	Jerry R. Mendell
ClinicalTrials.gov Identifier	NCT00428935	NCT03368742	NCT03375164	NCT03362502	NCT03333590	NCT02376816
Trial Title	Safety study of minidystrophin gene to treat Duchenne Muscular Dystrophy	Microdystrophin gene transfer study in adolescents and children with DMD (IGNITE DMD)	Systemic gene delivery clinical trial for Duchenne Muscular Dystrophy	A study to evaluate the safety and tolerability of PF-06939926 gene therapy in Duchenne Muscular Dystrophy	Gene transfer clinical trial to deliver rAAVrh74.MCK.GALGT2 for Duchenne Muscular Dystrophy	Clinical intramuscular gene transfer trial of rAAVrh74.MCK. microdystrophin to patients With Duchenne Muscular Dystrophy
Recruitment Status	Completed	Suspended (clinical hold)	Active, not recruiting	Recruiting	Active, not recruiting	Completed
Study Start Date	March 2006	6 December 2017	4 January 2018	23 January 2018	6 November 2017	March 2015
(Estimated) Study Completion Date	July 2010	March 2021	April 2021	26 August 2025	November 2021	September 2017
Intervention/Treatment	Biological: rAAV2.5-CMV-minidystrophin (d3990)	Genetic: SGT-001	Genetic: rAAVrh74.MHCK7 microdystrophin	Genetic: PF-06939926	Biological: rAAVrh74.MCK.GALGT2	Biological: rAAVrh74.MCK. microdystrophin
Enrollment	6 participants	16 participants	4 participants	15 participants	6 participants	2 participants
Patient Age	5–12 years	4–17 years	3 months to 7 years	4–12 years	4 years and older	7 years and older
Dose	Cohort 1: 2.0E10 vg/kg Cohort 2: 1.0E11 vg/kg	Ascending doses (quantitative value not reminded)	2.0E14 vg/kg in 10 mL/kg	Ascending doses (quantitative value not reminded)	Cohort 1: 5.0E13 vg/kg Cohort 2: 1.0E14 vg/kg	Cohort 1: 3E11 vg/single foot Cohort 2: 1E12 vg/single foot
AAV Serotype	AAV2.5	AAV9	AAVrh74	AAV9	AAVrh74	AAVrh74
Delivery Type	Intramuscular injection into biceps muscle	Intravenous injection	Intravenous injection into peripheral arm vein	Intravenous injection	Intravascular limb infusion	Intramuscular injection into Extensor digitorum brevis (EDB) muscle
Primary Outcome	Safety and tolerability [88]	Safety	Safety	Safety and tolerability	Safety	Safety
Secondary Outcome	Minidystrophin gene expression and muscle strength test [88]	No secondary outcome yet	Microdystrophin expression and muscle motility assessment	Minidystrophin gene expression, muscle strength and quality	GALGT2 gene expression and muscle motility assessment	Transgene expression
